# Aflatoxin M1 in Cow’s Milk: Method Validation for Milk Sampled in Northern Italy

**DOI:** 10.3390/toxins8030057

**Published:** 2016-02-26

**Authors:** Alberto Bellio, Daniela Manila Bianchi, Monica Gramaglia, Andrea Loria, Daniele Nucera, Silvia Gallina, Marilena Gili, Lucia Decastelli

**Affiliations:** 1 Istituto Zooprofilattico Sperimentale Piemonte, Liguria e Valle d’Aosta, via Bologna 148, Turin 10154, Italy; alberto.bellio@izsto.it (A.B.); monica.gramaglia@izsto.it (M.G.); andrea.loria@izsto.it (A.L.); silvia.gallina@izsto.it (S.G); marilena.gili@izsto.it (M.G.); lucia.decastelli@izso.it (L.D.); 2 Dipartimento di Scienze Agrarie, Forestali e Alimentari, Largo Braccini, 2, Grugliasco 10095, Italy; daniele.nucera@unito.it

**Keywords:** aflatoxin M1, milk, food safety

## Abstract

Aflatoxins (AFs) are mycotoxins produced by some species of *Aspergillus*. In dairy cows, ingested AFB1 is metabolized into carcinogenic AFM1 which is eliminated through milk, thus posing a risk for consumer health. Here we describe the set, validation, and application of screening (ELISA) and confirmatory (HPLC) tests carried out on milk samples collected through official control of mycotoxin levels in northern Italy over a three-year period (2012–2014). The limit of detection (LOD) was set at 5 ppt (ng/kg) and 2 ppt for ELISA and HPLC, respectively, and the limit of quantification (LOQ) was 10 ppt for confirmatory HPLC. A total of 1668 milk samples were analyzed: ELISA identified 36 (2.2%) positive milk samples that were subsequently confirmed by HPLC. The level of AFM1 in the positive samples ranged between 18 ± 2 and 208 ± 27 ppt. Of the total samples, only eight (0.5%) were found non-compliant with the EU regulatory limit (50 ppt; range 74 ± 10 to 208 ± 27 ppt). Use of ELISA and HPLC tests in series allows for high-volume analysis of samples, thus saving time and money while guaranteeing high analytical precision and accuracy.

## 1. Introduction

Many genera of molds growing on agricultural products produce toxic substances, generally called mycotoxins: some have mutagenic or carcinogenic effects, others are toxic for specific organs, and others still pose health risks owing to the effects they can have on an organism (e.g., vomiting or immunodeficiency) [1].

Mycotoxins are secondary metabolites of various different fungal species and they differ in chemical structure, biosynthetic origins, and biological effects. Clinicians classify mycotoxins as hepatotoxins, nephrotoxins, neurotoxins, immunotoxins, and so forth, whereas cell biologists classify them as teratogens, mutagens, carcinogens, and allergens. Aflatoxin, for example, is a hepatotoxic, mutagenic, carcinogenic, polyketide-derived *Aspergillus* mycotoxin [2]. Of the over 300 types of mycotoxins identified so far, aflatoxins (AFs) are the most extensively studied and one of most important classes from a public health perspective [3]. AFs figure among the most serious and well-known naturally occurring toxins in food and feed, with AFB1 being the most toxic and carcinogenic [4].

The first AFs were discovered in England in 1961 when a severe outbreak of turkey “*X*” disease resulted in the death of more than 100,000 birds. Peanut meal feed contaminated with a toxin produced by a filamentous fungus was found to be the source of the outbreak [5]. AFs are mainly produced by fungi of the genus *Aspergillus* (particularly *A. flavus*, *A. parasiticus* and rarely *A. nomius*) [6]. About 20 AFs belong to a larger group of toxic compounds called di-furanocoumarins, only four of which (AFB1, AFG1, AFB2, and AFG2) naturally contaminate foods. The hydroxymetabolites aflatoxin M1 (AFM1) and aflatoxin M2 (AFM2) can be present in milk and milk products when lactating cows or other mammals ingest contaminated feed [7]. It is estimated that about 1%–3% [8] to 6% [9] of AFB1 in feed is present as AFM1 in milk from within a few hours after the ingestion of contaminated meal up to two days after suspension of feeding the diet [10].

Due to its high hepatocarcinogenic potential, the level of AFM1 permitted in milk and dairy products is strictly regulated in the developed countries: the regulatory limit for AFM1 in milk and dairy products is 50 ppt in European countries and 500 ppt in the United States. Furthermore, because the biotransformation of carcinogens is generally slower in children than in adults, European Union (EU) legislation on food contaminants [11] fixes a more restrictive limit (25 ppt) for milk intended for consumption by nursing infants and children.

Robust analytical methods are needed to detect mycotoxins. The choice of which analytical process is most appropriate will take into account the target molecule, chemical features, complex matrix, timing of testing, and required limits of detection/quantification [12]. The methods for determining AFM1 can be classified into two main groups: chromatographic and immunochemical.

Immunochemical methods are used for rapid screening of AFs in various sample matrices. Several approaches have been developed for the determination of AFM1, but the method of choice is dictated by the type of matrix (fresh, stored, pasteurized milk, liquid or powdered milk, cheese). Many commercially available products use enzyme-linked immunosorbent assay (ELISA), TLC or HPLC [13].

Generally, chromatographic methods require extensive sample preparation steps and well-trained personnel; therefore, they are usually used for either confirmation of the results obtained from rapid screening tests or for accurate quantitative determination of mycotoxins. Immunochemical methods for rapid screening use specific antibodies with good sensitivity. Several immunochemical techniques, including ELISA, immunoaffinity column assays (ICA), sequential injection immunoassay (SIIA), and radioimmunoassay (RIA), have been developed for the determination of AFM1 in milk.

Here we describe the validation protocols for ELISA and HPLC tests for screening and confirmatory detection, respectively, of AFM1 levels in milk. ELISA was validated for use as a qualitative (screening) approach and HPLC as a quantitative (confirmatory) approach. Performance was evaluated on artificially spiked milk samples. Furthermore, the aim of the study was also to analyze a large number of milk samples from North Italy during 2012–2014. Finally, we wanted to determine whether the differences in AFM1 levels correlated with climatic conditions (*i.e*., mean temperature, humidity rate, and precipitation) known to be risk factors for AF contamination of silage.

## 2. Results

### 2.1. Validation of ELISA

ELISA performance and efficiency were evaluated. Analysis of specificity showed that the β error was ≤5%, confirming that the test is able to discriminate the analyte. The different incubation temperatures had no significant effect on assay performance, indicating that the test is sufficiently rugged. Finally, sensitivity was 1.00 (95% confidence interval CI 0.91–1.00).

### 2.2. Validation of HPLC

The HPLC method was in good agreement with the criteria stated in Commission Regulation (EC) No. 401/2006 [14]. The method was linear in the range of 0.75–25 ppb (pg/μL), corresponding to 0.006–0.2 ppb in matrix, indicating no interference by the food matrix and acceptable specificity. The tests to check repeatability and recovery are reported in Table 1 and were considered satisfactory according to internal requirements and parameters.

The limit of detection (LOD) and quantification (LOQ) were 0.002 ppb and 0.010 ppb, respectively. Method ruggedness was satisfactory at the tested conditions. Stability tests confirmed that stock solutions are stable at −18 °C for 18 months. Finally, method uncertainty was 13%.

**Figure 1:**
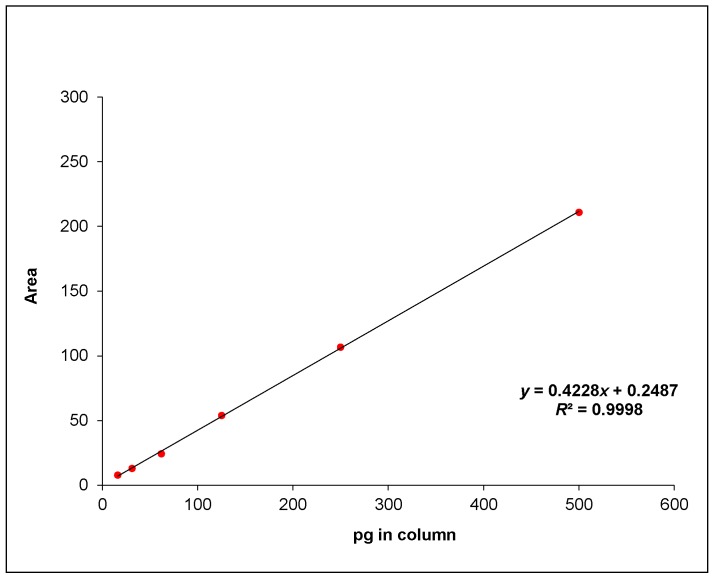
Curve obtained with spiked samples for the HPLC validation protocol.

Figure 1 shows the curve built for the validation protocol: the *R*^2^ was >0.99. This value needs to be reached in any analytical session in order to consider the test results acceptable.

### 2.3. Results of Sample Analysis

Between 2012 and 2014, a total of 1668 milk samples (548 in 2012; 625 in 2013; 495 in 2014) were collected throughout northwest Italy and delivered to the Milk Laboratory of the Istituto Zooprofilattico Sperimentale del Piemonte, Liguria and Valle d’Aosta (IZSTO) in Turin. Table 2 lists the surveys during which samples were collected by official veterinary local health services and by IZSTO researchers for study purposes.

Most samples, about 58% of the total number, were collected through the Raw Milk Automatic Self-Service Vending Machines Survey (2012–2014) to monitor the presence of AFM1 in milk for human consumption [15,16], followed by the Extraordinary Local Plan to survey AFM1 occurrence and climatic conditions (10.8%), the National Plan for Residue Detection (10.7%), the Regional Food Safety Plan (4.8%), the plan conducted on farms in compliance with Regulation EC 853/2004 requirements (2.2%), and the surveys for import-export activities (1.3%).

Of the total of 1,668 samples, 36 (2.2%) were found positive by ELISA and confirmed by HPLC (Table 3). According to the maximum acceptable limit fixed by European legislation, only eight samples were noncompliant (AFM1 levels >50 ppt). For these samples, quantitative HPLC showed a range in AFM1 from 74 ± 10 ppt to 208 ± 27 ppt (last column in Table 3).

Of these eight noncompliant samples, four were collected in 2012 and four in 2013: five samples were collected through the Raw Milk Vending Machines Survey, two through the Extraordinary AFM1 Survey, and one through the National Residues Plan. For the year 2012, four samples with AFM1 levels >50 ppt were collected: one in October and three in November; for the year 2013, two samples were collected in February and one each in May and July. All noncompliant samples were attributable to summer 2012 (*t* = 2.4; *p* = 0.04), whereas no statistical difference was detected between AFM1 concentrations (mean concentration 53.8 in 2012 *vs.* 43 in 2013).

**Figure 2:**
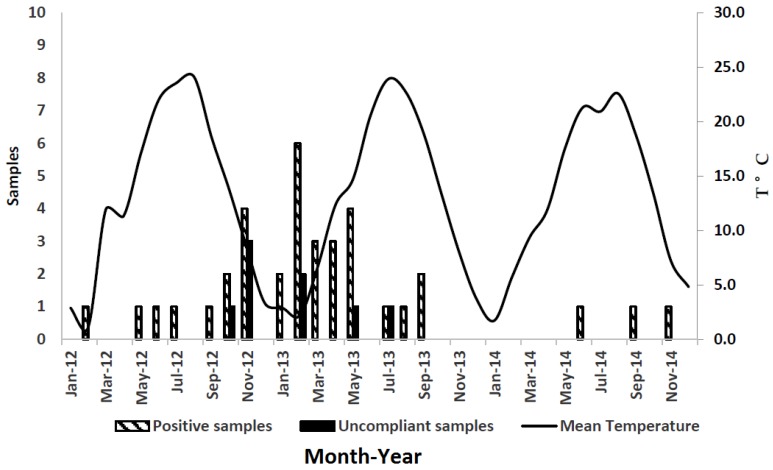
Mean temperature and number of positive samples from January 2012 to December 2014.

**Figure 3:**
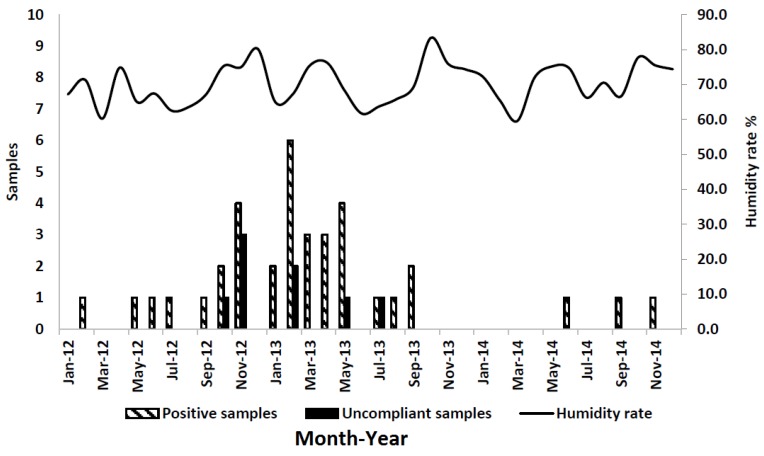
Humidity rate and number of positive samples from January 2012 to December 2014.

**Figure 4:**
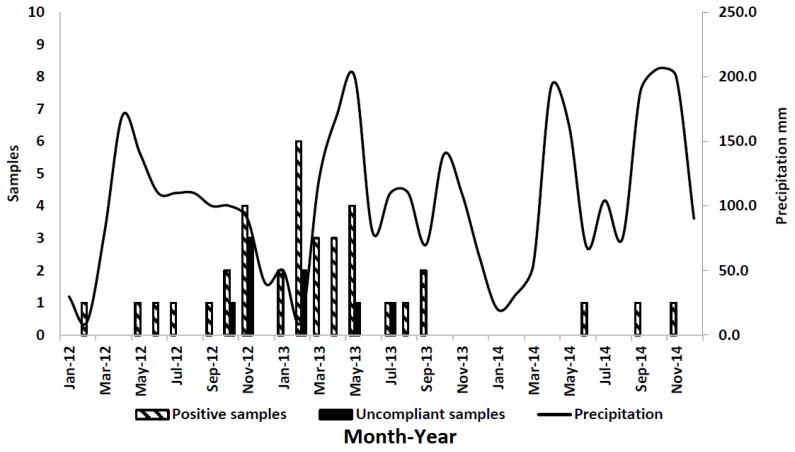
Precipitation and number of positive samples from January 2012 to December 2014.

Environmental conditions such as high humidity and drought promote AFB1 contamination in maize. Because these conditions predominate during summer in northwest Italy, farmers should make efforts to limit maize stress and *Aspergillus* development. Irrigation and fertilization are used to minimize the effect of drought and nutrition stress, respectively. Unfortunately, heat remained a major uncontrolled source of stress, although other unrecognized sources of stress may also have been present [17]. Average temperature, humidity rate (%), and precipitation data are reported in Figure 2, Figure 3 and Figure 4, respectively. The bar graphs show the number of positive and noncompliant samples by month of collection. Most positive samples (22/36; 61%) were collected between November 2012 and May 2013.

For statistical analyses, the AFM1-positive samples were divided into two groups: positive milk samples collected September 2012 through August 2013 and assumed to be associated with the climatic conditions of the previous summer (May–August 2012) (group 1), and, similarly, milk samples collected September 2013 through August 2014 (group 2) (Table 4). This was done in order to account for seasonal differences in climatic conditions across the two years: the average recorded temperature (May–August) for the summer of 2012 was 21.7 °C and that for the summer of 2013 was 20.5 °C; the precipitation index (mm) for the summer of 2012 was 117.5 mm and that for the summer of 2013 was 125.0 mm; finally, the relative humidity for the summer of 2012 was 64.4% and that for the summer of 2013 was 64.9%.

There was a statistically significant difference in the number of ELISA positive samples/months between the two groups (mean value 2.25 and 0.25, respectively; *t* = 3.6; *p* = 0.003); and all noncompliant samples had been collected in the summer of 2012 (*t* = 2.4; *p* = 0.04).

## 3. Discussion

The cumulative data presented here originate from surveys conducted throughout northwestern Italy between 2012 and 2014. Overall, the AFM1 contamination rate was 2.2% but less than 1% of milk samples of the total number were noncompliant with the EU limit. Previous studies reported a contamination rate of 14% in milk samples from southern Italy, though the AFM1 level was never above the permitted EU limit (range 0 to 40 ppt) [18]. Annual surveys on goat and sheep milk in Sardinia reported AFM1-positive rates of 0%–4% in sheep and 0%–13% in goat [19]. The low rates found for northwest Italy can be explained as a result of the implementation of own-check analyses. Mandatory for dairy cow milk producers, this system entails daily analyses of milk samples during high-risk periods to alert for the presence of AFM1 when detected at concentrations above the permitted level. If an alert has been signaled, the farmer must withdraw the products and suspend delivering milk to market.

Unsurprisingly, 61.1% of the AFM1-positive samples were collected during the two periods, November 2012–May 2013 and November 2013–May 2014, from cattle in northern Italy fed with maize silage of the previous summer, a season known as important as a source of mycotoxins [20].

Similarly, other studies have shown that AFM1 contamination is more likely to occur in milk produced during cold than warm seasons [21]. Bilandzic *et al.* (2010) observed that AFM1 concentration in milk was statistically higher during winter and spring than summer and autumn [22]. According to the weather records for our study area, the mean temperature was 21.7 °C and the rainfall amount was 117.5 mm for the summer of 2012 (May through August), whereas the mean temperature was slightly lower (20.5 °C) and rainfall was more abundant (125 mm) in 2013. Indeed, all noncompliant samples were collected in the period immediately following the summer of 2012 (*t*= 2.4; *p* = 0.04), whereas no statistical difference was detected between AFM1 concentrations (mean concentration 53.8 in 2012 *vs.* 43 in 2013).

Environmental conditions, such as temperature, humidity, and rainfall, as well as seasonal effects play an important role in feed contamination with AFB1 [23,24,25]. Poor storage conditions and practices during the ensiling process or when the silo is open for feeding can also lead to fungal contamination [26]. For these reasons, the most effective method to control AFM1 concentration in milk is by reducing the AFB1 contamination of raw materials and cattle feed through the application of Good Agricultural and Storage Practices [27,28]. Mycotoxin control largely depends on taking proper care during pre- and post-harvest conditions [29]. The use of fertilizers, pest controls, and fungal-resistant crops, as well as maintaining low moisture content and temperature during storage conditions can prevent fungal and mycotoxin contamination [30,31].

Because AFB1 contamination levels vary with year and climate, it may be useful to develop an AFB1 monitoring program that takes into account climatic conditions and pre-harvest maize quality during its growing season and that includes a specific survey on AFM1 detection in milk.

As concerns other matrices affected by AFs contamination, the HPLC method is more sensitive and specific, whereas the ELISA technique is cheaper and easier in laboratory practices [32]. The HPLC procedure is complicated and requires investment in expensive equipment and highly skilled technicians [33]. However, HPLC and other chromatographic methodologies also offer the advantage over ELISA techniques in that individual toxins can be quantified in the same analysis session [34]. The use of reliable and validated tests is essential for the official control of mycotoxin levels in food and feed. By reviewing and validating new methods for the determination of AFM1, researchers continue to refine extraction, purification, and quantification techniques in order to improve the reliability of the results concerning the impact of this compound in milk and meet the current demand for minimum waste generation. Evaluation of other techniques showed different sensitivity and specificity; however, these studies did not carry out parallel validation protocols [13]. Furthermore, the use of two different tests in series, as done in this study, underlines the useful features of both methods for providing excellent laboratory performance. Also, this protocol enables the National Health Services to collect and analyze large numbers of samples with high accuracy at reduced costs while ensuring analytical results that are both highly sensitive and specific. Though ELISA-based techniques are useful for rapid screening of mycotoxins, they can produce false-positive results and, on occasion, lack acceptable quantification accuracy; quantitative confirmatory analysis is therefore required.

## 4. Experimental Section

### 4.1. Sample Collection

**Figure 5:**
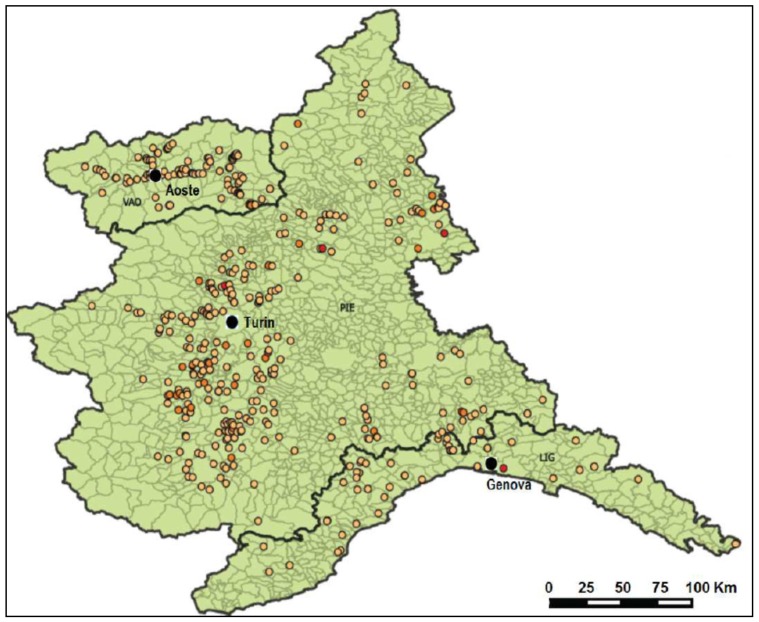
Spatial distribution of sampling sites.

The milk samples were delivered to the Milk Laboratory at Istituto Zooprofilattico Sperimentale del Piemonte, Liguria and Valle d’Aosta to determine the AFM1 level. These samples were collected throughout northwestern Italy (Figure 5) by the veterinary services of local health authorities for official controls and surveys and by IZSTO researchers for study purposes.

All samples were stored at 4 ± 2 °C until analysis within 24–48 h. An aliquot of each sample was frozen for use in other studies.

### 4.2. ELISA Screening

All samples were processed using a commercial competitive ELISA test (RIDASCREEN® Aflatoxin M1, R-Biopharm, Darmstadt, Germany) in a semi-quantitative method. As directed, a volume of 12 mL of milk was skimmed by centrifugation (3500 *g* for 10 min at +2/+8 °C) and the fat layer removed. ELISA plates were prepared with standards, samples, and a negative control. The negative control was a milk sample that resulted negative by HPLC; a positive control was a milk sample artificially spiked with 0.05 ppm (mg/kg) AFM1. A volume of 100 µL standard/sample was transferred into the well and incubated for 45 min at 20–25 °C. Liquid was discarded by capsizing the plate. Each well was washed 4 times with 250 µL of wash buffer supplied with the kit. Hereafter, 100 μL of AF-HPR (human haptoglobin-related protein) conjugate were added to each well; after incubation (15 min at 20–25 °C), the plate was washed 4 times with 250 µL wash buffer. A volume of 100 μL aflatoxin-HPR conjugate was dispensed in each well, and the plate was gently shaken and incubated (15 min at 20–25 °C). Finally, a volume of 50 μL stop buffer supplied with the kit was pipetted into each well, and the plate was read at 450 nm.

The tabulated OD values were entered in the R-Biopharm spreadsheet. Each analytical session reported the respective calibration curve for OD% values of samples and controls to quantify AFM1 levels. This test was used solely as a qualitative method: samples with AFM1 values ≥5 ppt were labeled “suspected positive” and processed by confirmatory HPLC. Samples with values <5 ppt (LOD) were considered “negative”.

### 4.3. HPLC Confirmatory Method

To confirm ELISA positive samples an HPLC method [35] was used and each sample was analyzed with two replicates. Sample preparation entailed a purification phase using 50 g of milk, previously skimmed by centrifugation at 3756 *g* for 15 min at 5 °C with immuno-affinity chromatography columns (IAC) (R-Biopharm AG, Darmstadt, Germany). First, the well was loaded with the sample and washed with 50 mL water. The analyte was eluted with 2 mL acetonitrile-methanol mixture (60:40 *v*/*v*). The flowthrough was dried with a nitrogen stream at 50 °C and suspended with 200 μL acetonitrile-methanol mixture (60:40, *v*/*v*) and 200 μL water. The suspension was then filtered on a 0.2 µm membrane filter. The chromatographic system was composed of an HPLC chromatograph system (Agilent 1200 series, Agilent Technologies, Santa Clara, CA, USA) with fluorimetric detection (FLD), λexcitation = 360 nm, λemission = 430 nm, column LiChrospher 60 RP SELECT B (Merck, Darmstadt, Germany) (250 × 4.6 mm, 5 μm) at 35 °C, isocratic elution with water-acetonitrile-methanol mixture (65:15:20, *v*/*v*/*v*), flow rate 1 mL/min, and injection volume 20 μL.

### 4.4. Validation of ELISA

The ELISA method was validated according to Commission Decision 2002/657/CE [36] concerning the performance of analytical methods and the interpretation of results. The protocol includes the following parameters: method specificity, ruggedness, sensitivity. Specificity was determined on 20 negative milk samples and 20 milk samples spiked with 40 ppt AFM1. To evaluate method ruggedness, the influence of 2 different incubation temperatures (25 °C and 30 °C) was evaluated with the approach of Youden [37]. Sensitivity was determined by testing 26 negative samples (<40 ppt) and 38 positive samples (>40 ppt) and was calculated using EpiTools epidemiological calculators: [38].

### 4.5. Validation of HPLC

The HPLC method was validated according to Commission Regulation (EC) No. 401/2006 [15]. Specificity was determined on 20 milk samples (range ∆*t* = R*t* ± 2.5%); linearity was calculated with a calibration curve by injection of standard in solvent at 0.8–25 ppb; precision was determined on milk samples spiked with 0.025–0.050–0.075 ppb or 0.5–1–1.5 times the permitted EU limit (0.050 ppb).

Each level of concentration was measured independently in 6 repeats. In each session, coefficient of variation (CV%) of repeatability for each level was calculated using analysis of variance (ANOVA) [39]; recovery was tested by external standardization and calculated as the average recovery for each level of concentration; limit of detection (LOD) and limit of quantification (LOQ) were calculated on 20 milk positive samples, in application of Regulation EC 401/2006; ruggedness was evaluated according to Youden’s index [37] and 8 experiments were performed on milk samples spiked with 0.050 ppb, introducing small changes during sample preparation and analysis. The variables were: IAC column type, IAC column washing buffer, elution volume, evaporation temperature, period, centrifugation speed and temperature; stability was determined by conservation at −18°C for 18 months of acetonitrile stock solution; finally, measurement of uncertainty was calculated by a bottom-up method [40] using coverage factor *K* = 2 and *n* = 2. Finally, 2 samples spiked with 0.05 ppb AF were tested in each analysis session in order to verify the accuracy and repeatability of analysis, following recovery control charts.

### 4.6. Statistical Analyses

Statistical analyses were performed using SPSS version 11.0 (SPSS Inc, Chicago, IL, USA, 2002). The number of positive samples/month among the samples attributable to silages prepared in summer 2012 was compared with those attributable to summer 2013. A similar analysis was performed on the number of noncompliant samples/month and the mean AF level in positive samples. After checking for the assumption of normality and homogeneity of variances, comparisons were performed using a *t*-test for unequal variances. Results with *p* < 0.05 were considered statistically significant.

## 5. Conclusions

The two methods validated in this study demonstrated good performance, being able to meet EU regulatory requirements and to detect AFM1 at the limits fixed by European legislation [15]. Ideally, a complete survey is based on screening and confirmatory testing of large numbers of samples. In this study, the use of ELISA for screening yielded rapid results with high sensitivity; subsequent confirmation by HPLC was particularly useful to detect the noncompliant milk samples.

The contamination rate (2.2%) in the milk samples indicates that AFM1 continues to pose a risk for human health; nevertheless, less than 1% of the samples had an AFM1 level >50 ppt. The permitted limits set by EU legislation are derived from studies on the effects of long-term exposure to AFM1, and this is to be considered as further protection for consumer safety [14].

The daily mean temperature during the maize-growing season was statistically correlated with the number of noncompliant milk samples, as all of them were collected in the summer of 2012. Monitoring temperature and humidity conditions may help to predict increases in AF content in feed and AFM1 levels in milk. Sampling plans carried out in Europe and Italy continuously monitor AFM1 levels in milk. The construction of databases can be a useful step to identify changes in AF levels in food for human and animal use and to manage possible emergencies that may occur.

## Figures and Tables

**Table 1:** Repeatability and recovery data.

Parameters	AFM1 Spike Doses		
	0.025 ppb	0.050 ppb	0.075 ppb
Repeatability (Coefficient of variation CV%)	9.7	7.6	7.0
Recovery (% ±standard deviation SD)	95 ± 7.8	93 ± 5.8	96 ± 5.8

**Table 2:** Samples collected through official surveys in northwest Italy (2012–2014).

Purpose	2012 (*N* = 548)	2013 (*N* = 625)	2014 (*N* = 495)	Total (*N* = 1668)	%
Raw Milk Vending Machine Survey	381	332	255	968	58.0
Study Purposes	6	35	142	183	11.0
Extraordinary AFM1 Local Survey	31	116	33	180	10.8
National Residues Plan	57	85	37	179	10.7
Regional Food Safety Survey	52	14	14	80	4.8
Tank Milk Survey (Regulation EC 853/2004)	3	31	3	37	2.2
Import-Export Survey	7	9	5	21	1.3
Other	11	3	6	20	1.2

**Table 3:** Aflatoxin M1 levels in milk samples.

Survey Year	Number of Samples (*N* = 1668)	Positive Samples (ELISA/HPLC) (*N* = 36)	Positive Samples (%) (% = 2.2)	Noncompliant with EU Limit (*N* = 8)	Noncompliant with EU Limit (%) (% = 0.5)	AFM1 Level in Noncompliant Samples (ppt) ^1^
2012	548	11	2.0	4	0.7	74 ± 10
						83 ± 11
						89 ± 12
						208 ± 27
2013	625	22	3.5	4	0.6	86 ± 11
						98 ± 13
						137 ± 18
						195 ± 25
2014	495	3	0.6	0	0.0	-

^1^ Data reported as concentration ± measurement uncertainty.

**Table 4:** Comparison between the number of AFM1-positive milk samples collected after the two summer periods (May–August 2012 and May–August 2013).

Month	Positive by ELISA (*N*)	Non-Compliant (*N*)	Month	Positive by ELISA (*N*)	Non-Compliant (*N*)
September 2012	1	0	September 2013	2	0
October 2012	2	1	October 2013	0	0
November 2012	4	3	November 2013	0	0
December 2012	0	0	December 2013	0	0
January 2013	2	0	January 2014	0	0
February 2013	6	2	February 2014	0	0
March 2013	3	0	March 2014	0	0
April 2013	3	0	April 2014	0	0
May 2013	4	1	May 2014	0	0
June 2013	0	0	June 2014	1	0
July 2013	1	1	July 2014	0	0
August 2013	1	0	August 2014	0	0
Total (*N*)	27	8	Total (*N*)	3	0
Mean	2.25	0.7	Mean	0.25	0

## Abbreviations

CI: Confidence Interval
EC: European Commission
ELISA: Enzyme-Linked ImmunoSorbent Assay
FLD: fluorometric detection
HPLC: High-Performance Liquid Chromatography
ppm: parts per million corresponding to mg/kg
ppb: parts per billion corresponding to μg/kg
ppt: parts per trillion corresponding to ng/kg
LOD: Limit of Detection
LOQ: Limit of Quantification
TLC: Thin-layer chromatography
UV: Ultraviolet
